# Occurrence, Source Apportionment and Health Risk Potential of Polycyclic Aromatic Hydrocarbons (PAHs) in Urban Soils from Thessaloniki City (Northern Greece): A Case Study

**DOI:** 10.3390/toxics14070582

**Published:** 2026-07-01

**Authors:** Anna Bourliva, Evangelia E. Golia, Evangelos Bakeas, Konstantinos Koukoulakis, Ioannis Papadopoulos

**Affiliations:** 1 Soil Science Laboratory, School of Agriculture, Faculty of Agriculture, Forestry and Natural Environment, Aristotle University of Thessaloniki, University Campus, 54124 Thessaloniki, Greece; egolia@agro.auth.gr (E.E.G.); papadopoulosjohn28@gmail.com (I.P.); 2 Environmental Chemistry Laboratory, Department of Chemistry, National and Kapodistrian University of Athens, 15784 Zografou, Greece; bakeas@chem.uoa.gr (E.B.); kkoukoulakis@chem.uoa.gr (K.K.)

**Keywords:** urban soils, PAHs, pyrogenic sources, traffic emissions, Thessaloniki

## Abstract

Urban soils act as sinks for polycyclic aromatic hydrocarbons (PAHs) indicating the intensity of the anthropogenic load, while potential environmental and human health concerns may arise. In the present study, the concentrations, spatial distribution, source apportionment and potential health risks of 16 priority PAHs were investigated in urban soils from the city of Thessaloniki, Northern Greece. Surface soil samples were collected from 19 locations characterized by different land uses and traffic conditions. The total levels of the 16 PAHs exhibited substantial variability, with a range of 14.09–1565.4 μg kg^−1^, reflecting heterogeneous contamination patterns across the city. PAH profiles were dominated by high-molecular-weight compounds (4–6 rings) accounting for over 80% of the total PAHs. Diagnostic molecular ratios highlighted pyrogenic sources, verifying that high-temperature combustion processes dominated the PAH inputs in the urban soils from Thessaloniki city. The factor score plot made prominent the presence of localized contamination hotspots in areas characterized by intense and continuous traffic activity, spotlighting vehicular traffic emissions and transport-related activities as primary sources of PAHs in the study area. Carcinogenic risk assessment based on the BaP-EQ approach indicated acceptable risk levels for most of the sampled soils, although limited localized hotspots with elevated carcinogenic risk were identified. This study provides important baseline information for understanding PAH contamination in urban environments and supports the development of targeted pollution mitigation and environmental management strategies.

## 1. Introduction

Polycyclic aromatic hydrocarbons (PAHs) constitute a broad group of organic pollutants mainly produced by the incomplete combustion of organic matter, including fuels [1]. PAHs have been internationally recognized as substances posing a high risk in terms of environmental and health hazards [2]. Due to their chemical structure, characterized by multiple condensed aromatic rings, PAHs exhibit low water solubility and high lipophilicity, properties that favor their accumulation in soil, especially in its organic matter [3]. Soil thus acts as a primary sink and reservoir for PAHs, especially in areas of intense human activity [4].

Kumar et al. [5] explain in their research how PAHs are directly linked to urban, industrial, and agricultural activities, such as vehicle traffic, the burning of fossil fuels and biomass, the use of sewage sludge, and atmospheric deposition of pollutants. In urban soils, PAH levels are often higher because of constant exposure to exhaust fumes, industrial emissions, and asphalt materials [6]. In agricultural soils, pollution is mainly related to irrigation, the use of organic residues, and farming practices [7]. PAHs persist in the soil for a long time, as they are compounds that are resistant to biodegradation, which increases the risk of their transfer to the food chain [8]. Atmospheric transport followed by deposition represents the dominant pathway by which PAHs enter soils [9]. After their release from combustion sources such as vehicle exhaust, residential heating, and industrial activities, PAHs are dispersed by air masses and subsequently transferred to the soil through: dry deposition, involving the gravitational settling of particle-bound PAHs onto soil surfaces, and wet deposition, whereby PAHs are scavenged from the atmosphere by precipitation (rain, snow, and fog) and delivered to the soil [10]. This process explains the widespread occurrence of PAHs even in remote or rural soils, far from direct emission sources, and highlights the role of long-range atmospheric transport in diffuse soil contamination.

Their environmental importance derives not only from their toxicity, however, but also from their potential to affect the functioning of soil ecosystems, microbial activity, and soil fertility [11]. Recent studies have further demonstrated that aromatic compounds, including PAHs, can alter microbial community structure and influence nitrogen transformation processes, highlighting the complex interactions between organic contaminants and soil biogeochemical cycling [12,13]. Furthermore, human exposure to PAHs through food, inhalation of particles, or direct contact with contaminated soil has been linked to serious health effects, including carcinogenesis and genetic damage [14]. Montano et al. [15] described in their narrative review both the bioaccumulation and metabolization of PAHs in humans, focused on female fertility.

PAHs most commonly detected in soils are primarily characterized by greater stability and low volatility [16]. Those found at the highest concentrations in urban and agricultural soils are predominantly medium- to high-molecular-weight compounds (3–6 aromatic rings), which exhibit strong persistence and affinity for soil organic matter. Ukalska-Jaruga and Smreczak [17] investigated the influence of both fulvic and humic acids along with humins, found on soil organic matter, on PAH availability and persistence in soils. These include phenanthrene, anthracene, fluoranthene, pyrene, chrysene, benzo[a]anthracene, benzo[b]fluoranthene, benzo[k]fluoranthene, benzo[a]pyrene, indeno [1,2,3-cd]pyrene, dibenzo[a,h]anthracene, and benzo[g,h,i]perylene. These compounds are commonly reported in contaminated urban and agricultural soils worldwide and are often included in the priority pollutant lists of regulatory agencies due to their toxicity, persistence, and carcinogenic potential [18].

Among them, fluoranthene and pyrene are widely recognized as combustion-derived markers, as they are formed in high yields during the incomplete combustion of fossil fuels and biomass. Fabiańska et al. [19] explain how certain geochemical markers along with PAHs found in solvent extracts are from machines using diesel fuels. Their ubiquitous presence in urban soils reflects the dominant influence of traffic emissions, residential heating, and industrial combustion processes [20]. In contrast, benzo[a]pyrene is frequently used as a key indicator of carcinogenic risk, owing to its strong mutagenic and carcinogenic properties and its well-documented association with adverse human health effects [21]. Elevated concentrations of benzo[a]pyrene in soils are therefore considered a reliable proxy for assessing the severity of PAH-related health hazards in urban and peri-urban environments [22].

Therefore, studying the presence, origin, and behavior of PAHs in soil is a critical area of environmental science and soil science, contributing to the design of strategies for pollution prevention and remediation. Despite the environmental significance of Thessaloniki, the second largest city in Greece and a major transportation and commercial hub in the eastern Mediterranean, information regarding the occurrence and source characteristics of PAHs in its urban soils remains limited. Given the intense urbanization, heavy traffic density, and transportation-related activities within the city, it was hypothesized that urban soils would exhibit heterogeneous PAH accumulation patterns, with elevated concentrations in areas subjected to intense anthropogenic pressure, and that combustion-related emissions would constitute the dominant source of contamination. The objectives of the present study were therefore: (a) to evaluate the contents of PAHs in urban soils, (b) to examine their potential sources, (c) to point out their spatial distribution and appraise the contribution of anthropogenic activities and (d) to estimate the potential impacts on human health. The study further aims to provide baseline information for Mediterranean urban environments and contribute to the broader understanding of PAH accumulation and source dynamics in traffic-dominated cities.

## 2. Materials and Methods

### 2.1. Study Area and Soil Sampling

**Figure 1:**
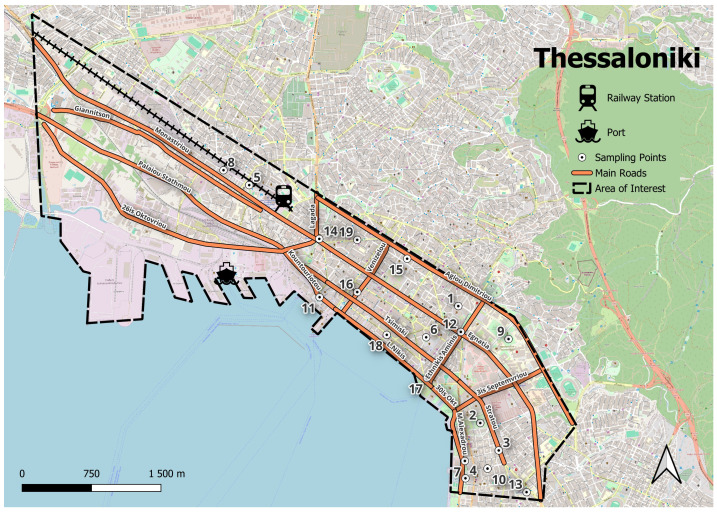
Study area and sampling sites of urban soils in the city of Thessaloniki.

During the years 2023–2024, a total of 38 surface (0–20 cm) soil samples (19 per year) were collected from selected locations in the city of Thessaloniki, the second largest city of Greece, ensuring a geographically uniform sampling within the urban area (Figure 1). The same sampling sites were revisited during both sampling campaigns to ensure spatial consistency and improve dataset robustness. Additionally, the primary objective of the study was to evaluate the spatial distribution, source apportionment, and health risks of PAHs in urban soils rather than the temporal variability. Sampling was conducted during the dry season (June–July) in both sampling years in order to minimize the influence of short-term meteorological variability on soil PAH concentrations.

The soil sampling for urban polycyclic aromatic hydrocarbon (PAH) assessment was conducted by collecting a composite sample consisting of five individual subsamples within a 1.5 m radius area, ensuring adequate spatial representation of heterogeneous urban contamination [23]. Each subsample was obtained from the 0–20 cm topsoil layer using dedicated stainless-steel corers and thoroughly cleaned tools to prevent cross-contamination. The subsamples were homogenized in a clean, inert surface tray to generate a single representative composite sample according to established environmental sampling protocols. All sampling equipment was decontaminated between sites to maintain analytical accuracy. The composite soil was transferred into pre-cleaned amber glass containers with PTFE-lined caps to minimize PAH adsorption and photodegradation [24]. Samples were immediately labeled, shielded from light, and stored under cooled conditions during transport to the Environmental Chemistry Laboratory of the Chemistry Department, of the National and Kapodistrian University of Athens. Upon arrival, samples were refrigerated until the extraction and subsequent GC-MS determination of target PAHs following standard analytical procedures. This multi-point compositing strategy enhances representativeness and reduces the microscale variability characteristic of urban soils. Detailed field notes documenting sampling depth, environmental conditions, and handling steps were maintained to support data integrity and reproducibility [25].

### 2.2. Analytical Procedure

The 16 PAHs analyzed in this study were Napthalene (Nap), acenaphthylene (Ace), acenaphthene (Acy), fluorene (Flu), phenanthrene (Phe), anthracene (Ant), fluoranthene (Fla), pyrene (Pyr), chrysene (Chr), benzo[a]anthracene (BaA), benzo[b,k]fluoranthene (BbkF), benzo[a]pyrene (BaP), indeno [1,2,3-cd]pyrene (InP), dibenzo[a,h]anthracene (DahA), and benzo[g,h,i]perylene (BghiP).

The procedure for PAH extraction and analysis was based on USEPA method 3550C [26] and that described by Iwegbue et al. [27] with some modifications, which included a number of steps. Prior to extraction, 4 g of the sample was spiked with the internal standards phenanthrene-d10 and perylene-d12 (Sigma-Aldrich, St. Louis, MO, USA). The sample was extracted in triplicate with 30 mL DCM HPLC grade in an ultrasonic bath for 20 min. The extract was concentrated using rotary evaporation to an approximate volume of 2 mL. Then, 10 mL of hexane HPLC grade were added. The solution was concentrated again to 2 mL and a second addition of 5 mL hexane was performed. The final extraction was concentrated by a rotary evaporator to nearly 2 mL for further cleanup.

A 20 cm × 7 mm i.d. glass column chromatograph was used. The column was packed from bottom to top with glass wool (Sigma Aldrich), 1 g of anhydrous sodium sulfate High Purity grade >99.5% (Panreac, Barcelona, Spain) and 1 g of silica gel High Purity grade (Sigma Aldrich). Initially and before sample loading, the column was activated with the addition of 10 mL of hexane. After this step, the sample (nearly 2 mL) was placed on the top of the column). The elution for PAHs was performed using 8 mL hexane and then 10 mL of a CH_2_Cl_2_: n-hexane 60:40 mixture. The eluted fraction was evaporated under a flow of nitrogen steam and was finally adjusted to 0.5 mL.

An Agilent 6890 N GC (Agilent Technologies, Santa Clara, CA, USA) equipped with an Agilent 7683B Injector (Agilent Technologies, Santa Clara, CA, USA), a HP 5 MS capillary column (30 m × 0.25 mm i.d) (Agilent Technologies, Santa Clara, CA, USA) coated with 5% Phenyl-methylpolysiloxane (film thickness) 0.25 μm, and an Agilent 5975B mass selective detector (MSD) were used for PAH determination. A total of 1 μL of each sample was injected in the pulsed splitless mode by an auto sampler (Agilent 7673 A). The injector temperature was set at 280 °C and the ion source temperature at 230 °C. The oven operating temperature ranged from 65 to 320 °C at a rate of 15 °C/min. The quantification was performed using the selected ion monitoring (SIM) mode.

Six point calibration curves (0.5–25 ng) were prepared and the r^2^ was >0.99 for all the analytes. The QA/QC included a method blank sample and matrix-spiked and duplicate samples. The recoveries of the studied PAHs ranged from 76% to 125% using spiked samples. Recoveries slightly exceeding 100% are attributed to matrix-induced signal enhancement and normal analytical variability, phenomena commonly observed in trace organic analysis, and are considered acceptable given the satisfactory precision of the method. The method LOQs were determined as 10 times the signal-to-noise level of the blank soil sample, and they were evaluated by preparing a standard solution of PAHs at this level. The LOQ values of the method ranged from 0.004 to 0.17 ng.

### 2.3. Source Apportionment and Multivariate Analysis

In order to investigate the dominant sources of PAHs in urban soils from the city of Thessaloniki, commonly used molecular diagnostic ratios were applied based on selected PAH pairs with similar physicochemical properties but different source origins [28,29]. Moreover, PCA (Principal Component Analysis) was carried out using Varimax rotation to make interpretation easier by focusing variable loadings on fewer factors, keeping just those with eigenvalues greater than 1. The Kaiser–Meyer–Olkin (KMO) measure of sampling adequacy, which was 0.779, and Bartlett’s Test of Sphericity, which resulted in a chi-square value of 851.2 (df = 105, *p* < 0.001) validated the data’s eligibility for PCA by showing enough correlations among variables. Multivariate statistical analyses were performed using SPSS software (version 29.0, IBM Corp., Armonk, NY, USA).

### 2.4. Carcinogenic Risk Assessment Using BaP-EQ Concentrations

Benzo[a]pyrene (BaP-EQ) was used as an indicator compound based on its well-known carcinotoxicity among the PAHs. To evaluate the risk assessment, Nisbet and LaGoy [30] developed toxicity equivalency factors (TEFs) that convert PAH contents into BaP equivalent concentrations (BaP-EQ) using the following formula:

$$BaP-EQ=\sum_{i=1}^{n}{PAH}_{i}\times {TEF}_{i}$$

where PAH_i_ is the concentration of each PAH in μg kg^−1^ and TEF_i_ is the toxic equivalence factor of each PAH (Table S1).

## 3. Results and Discussion

### 3.1. PAH Concentrations in Urban Soils

The concentrations of the 16 U.S. Environmental Protection Agency (USEPA) priority PAHs (in μg kg^−1^) were determined in urban soils from the city of Thessaloniki and the results are summarized in Table S2, while their descriptive statistics are given in Table 1.

The total concentration of PAHs (Σ_16_PAHs) ranged from 14.09 to 1565.4 μg kg^−1^ with an average value of 586.4 μg kg^−1^. The seven carcinogenic PAHs (Σ_7_PAHs)’ concentrations ranged from 7.6 to 977.3 μg kg^−1^ (mean of 365.5 μg kg^−1^), while they accounted, on average, for 59.3% of the total PAHs. The Σ_16_PAHs exhibited a wide range, with maximum levels more than 100 times higher than the minimum ones, indicating heterogeneous contamination. Overall, the Σ_16_PAHs in 74% of the soil samples were <1000 μg kg^−1^, indicating that multiple anthropogenic activities such as vehicular traffic, residential combustion, and urban industrial activity mainly influence PAH concentrations in urban soils. The highest values of Σ_16_PAHs were recorded in sample 5 (1565.4 μg kg^−1^), followed by samples 17 (1357.9 μg kg^−1^), and 8 (1190.5 μg kg^−1^) collected from areas with high traffic flow and continuous vehicular activity. In addition, samples 5 and 8, which were nearby the railway station area, represent a complex emission environment, which further explains the elevated PAH levels. Moderate to elevated concentrations were also observed in samples from commercial and residential areas, suggesting that PAH contamination extends beyond high traffic zones.

In order to assess soil pollution, Maliszewska-Kordybach [31] proposed a classification for soil contamination into four levels based on Σ_16_PAHs: uncontaminated soils (<200 μg kg^−1^); slightly contaminated soils (200–600 μg kg^−1^); contaminated soils (600–1000 μg kg^−1^); and heavily contaminated soils (>1000 μg kg^−1^). According to this classification, 26% of the soil samples were characterized as heavily contaminated, and 16% as contaminated, while 32% were considered as uncontaminated.

The obtained PAH levels were compared with those reported for urban soils worldwide. In general, the recorded PAH concentrations were comparable to moderately contaminated urban environments. For instance, urban soils in Tarragona [32] and Santiago de Compostela [33] in Spain exhibited values of the same order of magnitude. Similarly, Qu et al. [34] reported comparable PAH levels (460 μg kg^−1^) for urban park soils in Beijing, suggesting comparable urban emission intensities and anthropogenic loads. Also, Škrbić et al. [35] reported mean Σ_16_PAH values of 298 μg kg^−1^ for urban soils in Novi Sad, Serbia.

In contrast, significantly higher PAH concentrations have been reported in areas associated with heavy industrial inputs. For example, urban soils in Krakow, Poland exhibited significantly higher PAH levels (up to 18,000 μg kg^−1^), reflecting intense industrial activity and long-term accumulation of combustion-derived pollutants [36]. Similarly, Morillo et al. [37], in a comprehensive study of urban soils from three European cities, reported elevated Σ_16_PAH ranges in Glasgow (up to 51,822 μg kg^−1^) attributed to intense industrial and traffic emissions, while notable lower levels were recorded in Torino and Ljubljana, but were still higher than the obtained mean values of this study. In Eastern European megacities such as Saint Petersburg, Σ_16_PAHs exhibited values up to 8100 μg kg^−1^, with high HMW fractions and discernible land-use differences in contamination [38]. Similarly, urban soils in other global regions also demonstrate comparable or higher PAH levels. For example, in urban soils from Sanandaj, Iran, Σ_16_PAH concentrations ranged from 126.44 to 2460.87 μg kg^−1^ with a mean of approximately 850.81 μg kg^−1^ [39]. Additionally, urban soils from Xi’an (China) showed Σ_16_PAHs ranging from 390.6 to 10,652.8 μg kg^−1^ with a relatively large variation across land uses, and carcinogenic PAHs contributing significantly to total loadings [40]. In Beijing, Σ_16_PAH concentrations have been reported from 93 to 13,141 μg kg^−1^ in urban soils [41]. Although the maximum PAH levels surpass the obtained PAH values in Thessaloniki, the mean concentration of 1228 μg kg^−1^ lies within the Thessaloniki range, highlighting that large cities with intensive traffic and industrial activities can produce comparable contamination levels despite differing degrees of urbanization.

### 3.2. PAH Profiles in Urban Soils

**Figure 2:**
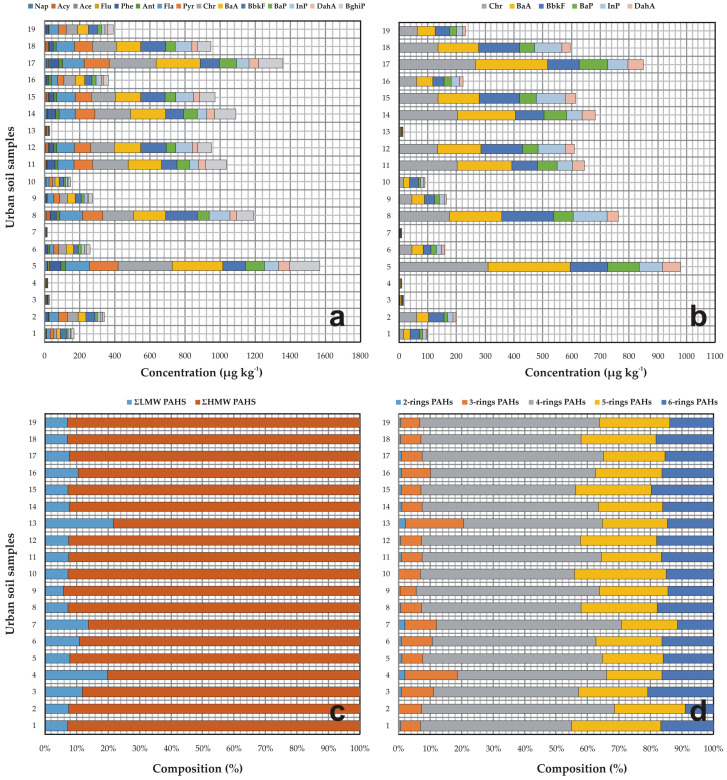
PAH fingerprinting results showing: (**a**) PAH concentrations (μg kg^−1^), (**b**) carcinogenic PAH concentrations (μg kg^−1^), (**c**) LMW vs. HMW composition (%) and (**d**) ring-number compositional profiles (%) in urban soils from the city of Thessaloniki.

The compositional profiles of PAHs in urban soils from the city of Thessaloniki showed clear and consistent patterns across the sampling sites, as illustrated in Figure 2.

Among the quantified PAHs, chrysene (Chr), benzo[a]anthracene (BaA), benzo[b,k]fluoranthenes (B(b,k)F), pyrene (Pyr), and fluoranthene (Fla) were the most abundant compounds, with mean values of 98.99 μg kg^−1^, 97.07 μg kg^−1^, 69.16 μg kg^−1^, 61.61 μg kg^−1^, and 58.76 μg kg^−1^, respectively. Carcinogenic PAHs also exhibited significant variability and formed an important fraction of the total PAH burden. In particular, chrysene (Chr) and benzo[a]anthracene (BaA) dominated in the sampled soils (Figure 2b). For instance, Chr reached up to 309.8 μg kg^−1^, and BaA up to 285.8 μg kg^−1^, indicating the dominance of combustion-related PAHs in urban soils. Pyrene and fluoranthene were also prominent compounds, with values up to 162.9 μg kg^−1^ and 132.5 μg kg^−1^, respectively, both contributing to a major share of the total PAHs, especially in sites with elevated contamination levels.

Benzo[b,k]fluoranthenes exhibited elevated concentrations reaching up to 180.1 μg kg^−1^, indicating localized hotspots. On the other hand, BaP, a key marker of carcinogenic risk, and DhA, despite their lower concentrations (0.53–108.92 μg kg^−1^ and 0.59–62.18 μg kg^−1^, respectively), consistently contributed to the toxicologically relevant fraction of Σ_16_PAHs. Low-molecular-weight individual PAHs, such as acenaphthylene, acenaphthene, fluorene, and anthracene, exhibited relatively lower concentrations and contributed less to Σ_16_PAHs.

Across all samples, HMW (4–6 rings) PAHs overwhelmingly dominated, with their combined contribution exceeding 80–90% of total PAHs, particularly in sites characterized by higher overall contamination (Figure 2c). This finding reflects the strong persistence of HMW PAHs in soils and their preferential association with particulate matter derived from combustion processes [34,42]. The predominance of HMW PAHs in Thessaloniki soils parallels findings in diverse urban environments, where traffic emissions, domestic heating, and industrial activities collectively shape PAH patterns [43,44]. In contrast, LMW-PAHs (2–3 rings) contributed a smaller fraction of total PAHs, generally accounting for less than 10% of ΣPAHs in most of the sampled soils. Phenanthrene was detectable in all sampled soils; however, its relative contribution was limited compared to that of HMW-PAHs. The lower abundances of LMW-PAHs are indicative of their higher volatility and susceptibility to degradation and redistribution in the urban environment.

Further classification of PAHs according to their ring number revealed a clear dominance of four-ring PAHs, followed by five- and six-ring compounds (Figure 2d). Four-ring PAHs represented the largest fraction of Σ_16_PAHs across the study area, in line with previous studies in multiple urban agglomerations [34,45,46,47]. Additionally, five-ring PAHs, such as B(b,k)F and BaP, also contributed significantly, particularly at sites influenced by intense urban activity. On the other hand, six-ring PAHs were consistently detected but contributed a smaller proportion to Σ_16_PAHs due to their generally lower concentrations.

In contrast, two-ring PAHs (naphthalene) and three-ring PAHs (phenanthrene and anthracene) were less dominant, particularly when Σ_15_PAHs (excluding naphthalene) were considered. However, the inclusion of naphthalene in Σ_16_PAHs increased the relative contribution of low-ring PAHs at selected sites, highlighting the influence of volatile PAHs on the total PAH burden when included.

These compositional characteristics are consistent with urban soils impacted primarily by combustion-related sources, such as vehicular traffic and residential heating, and provide a robust basis for subsequent discussion of PAH sources and carcinogenic risk.

### 3.3. Source Identification of PAHs in Urban Soils

#### 3.3.1. Diagnostic Ratios and Cross Plots

The identification of potential sources of PAHs was made based on different isomeric diagnostic ratios, which provide source identification in relation to the distribution of PAHs in urban soils, and the results are summarized in Table 2.

The Fla/(Fla + Pyr) ratio is one of the most widely used indicators for distinguishing petrogenic from combustion-derived PAHs. In the present study, values of this ratio were predominantly above 0.4, with 63% of the sites approaching or exceeding 0.5, thresholds commonly attributed to fossil fuel and biomass combustion rather than unburned petroleum inputs. Similar values have been reported for urban soils influenced by vehicular traffic and residential heating in European [35] and Asian [42,48] cities, reinforcing the interpretation that high-temperature combustion processes are dominating the PAH inputs in the urban soils from Thessaloniki city.

Consistent with this finding, Ant/(Ant + Phe) ratios in Thessaloniki soils were generally greater than 0.1, indicating pyrogenic sources. Specifically, the ratio values ranged from 0.12 to 0.48 with an average of 0.30. Phenanthrene typically dominated over anthracene, reflecting the greater thermodynamic stability of phenanthrene, and the observed ratios nonetheless point to combustion rather than petrogenic contamination. Comparable Ant/(Ant + Phe) values have been reported for urban soils impacted by traffic emissions and domestic fuel combustion, further supporting a combustion-driven PAH signature [46,49,50,51].

The BaA/(BaA + Chr) ratio exhibited values greater than 0.35 (a range of 0.41–0.70) with an average of 0.53, suggesting the role of combustion at all sites. Elevated BaA/(BaA + Chr) ratios have been linked to vehicular exhaust emissions in numerous urban soil studies [50,51], and the dominance of four-ring PAHs in the studied urban soils from Thessaloniki is consistent with this interpretation.

The InP/(InP + BghiP) ratio further refines the source attribution by distinguishing between various fuel uses. The ratio values were within the 0.2–0.5 range, suggesting a dominant influence of petroleum combustion, particularly from vehicular traffic. Higher values were recorded in 26% of the samples, probably reflecting localized contributions from residential heating, especially during colder periods when biomass or mixed fuels are used. Similar IP/(IP + B(ghi)P) ranges have been reported in urban soils from multiple European cities, where traffic emissions constitute a major source of PAHs [35,51].

**Figure 3:**
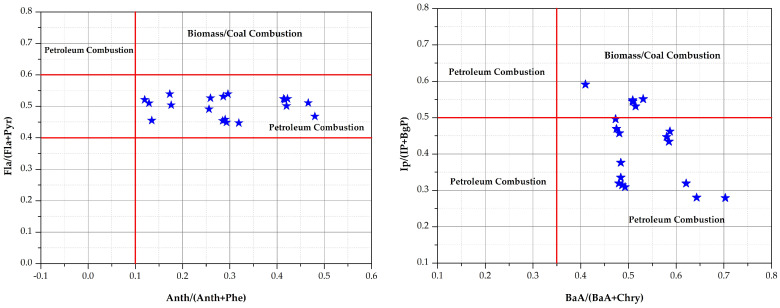
Ratio diagnostics of PAHs in soil samples from the city of Thessaloniki.

To corroborate these findings, the cross plot of ANT/(ANT + PHE) vs. FLA/(FLA + PYR) provides further insight into the dominant sources of PAHs in the urban soils of the city of Thessaloniki (Figure 3). All data points cluster within the petroleum combustion field, suggesting that vehicular traffic emissions represent the dominant source of PAHs across the study area. The absence of samples in the petrogenic filed further indicates that unburned petroleum inputs play a negligible role in PAH accumulation in Thessaloniki soils.

Further source discrimination is provided by the IP/(IP + BgP) vs. BaA/(BaA + Chr) cross plot (Figure 3). The majority of the samples fall within the petroleum combustion field, while a subset of samples plots near or above the IP/(IP + BgP) threshold of 0.5, reflecting a mixed contribution from biomass and coal combustion. No samples fall within the petrogenic region of the diagram, confirming again the negligible influence of unburned petroleum sources. The agreement between the two diagnostic cross plots strengthens the conclusion that urban soil PAHs are mainly derived from combustion processes, dominated by vehicular emissions with secondary inputs from biomass and coal combustion.

Nevertheless, it is important to acknowledge the inherent limitations of diagnostic ratios when applied to soils. Urban soils represent integrative environmental matrices that can accumulate PAHs for long periods of time, during which atmospheric transport, weathering, volatization, photodegradation, microbial degradation, and mixing of emissions from multiple sources may modify the original PAH signatures and consequently affect diagnostic ratio values. Therefore, diagnostic ratios should be interpreted cautiously and used primarily as indicative rather than definite source tracers. In the present study, the strong dominance of HMW-PAHs, the prevalence of 4–6 ring compounds, and the elevated concentrations of combustion-associated monomers collectively support the results of the diagnostic ratios and suggest that traffic emissions and other combustion-related activities are the primary contributors to PAHs in Thessaloniki’s urban soils.

#### 3.3.2. Multivariate Apportionment of PAHs

In order to further elucidate the potential emission sources of PAHs in the sampled urban soils from the city of Thessaloniki, Principal Component Analysis (PCA) was applied to the whole dataset, and the resulting principal components (PCs) are presented in Table 3. In total, two PCs were extracted, explaining 97.9% of the total variance.

PC1 is characterized by high loadings of mixed 3–5 ring PAHs including Nap (0.898), Ace (0.924), Fl (0.992), Phe (0.964), Pyr (0.855), Chr (0.927), BaA (0.895), BaP (0.898), DhA (0.897), and BP (0.907) and moderate loadings for Ant (0.783) and Fla (0.731), commonly associated with combustion-derived emissions. The predominance of compounds such as Pyr, Chr, BaA and BaP suggest an important contribution from pyrogenic processes, including vehicular traffic and fossil fuel combustion [29,48]. The co-presence of low-molecular-weight (LMW) and carcinogenic high-molecular-weight (HMW) PAHs suggests mixed fresh and aged pyrogenic inputs which are consistent with persistent accumulation in urban soils. In any case, since several combustion sources produce similar PAH assemblages, the component should be interpreted as representing a mixed combustion source rather than a unique emission category.

On the other hand, PC2, linked to a specific traffic/diesel or mixed combustion source, is characterized by strong loadings for Acy (0.996), B(b,k)F (0.840) and InP (0.872), which are mainly associated with high-temperature fossil fuel combustion, particularly diesel exhaust. Moreover, moderate loadings for Fla (0.667), Ant (0.581) and Pyr (0.508) further support a combustion-related origin. Nevertheless, source attribution based solely on PCA should be interpreted with caution because similar PAH profiles may rise from multiple combustion-related activities. Therefore, this component is more appropriately described as reflecting localized combustion-related inputs with a possible contribution from diesel-powered transport sources. Overall, the PCA results corroborate the diagnostic ratios suggesting that the PAHs in the sampled urban soils are predominantly derived from pyrogenic sources, with vehicular traffic inputs being the primary contributor with minor additional contributions from other combustion processes.

**Figure 4:**
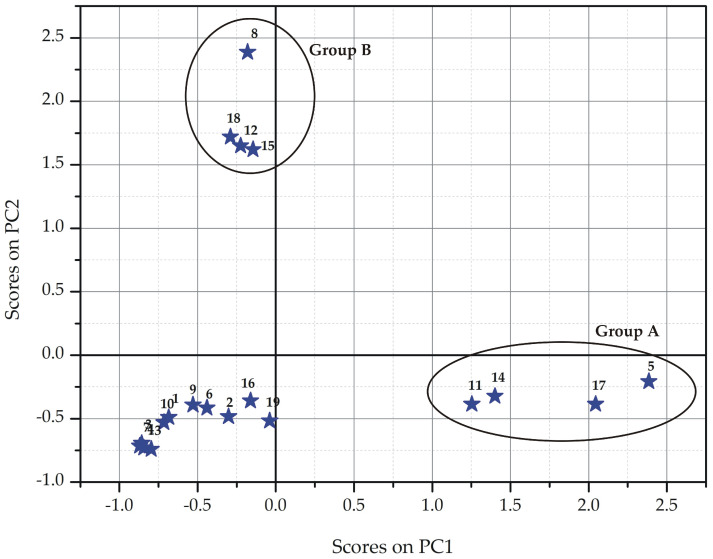
PCA score plot of soil samples from the city of Thessaloniki.

The factor score plot (Figure 4) suggested the spatial variability of PAH sources across the sampling sites across Thessaloniki. Most samples cluster in a region with relatively lower contributions from both the identified PAH sources, suggesting background urban contamination levels. In contrast, a distinct group of samples (Group A with sites 5, 11, 14, and 17) reflect strong influence from traffic-related sources corresponding to locations where high PAH levels were recorded (Table S2) and are clearly associated with areas of intensified anthropogenic activity. These locations include areas situated near major transportation routes and road intersections (Figure 1), which are strongly affected by intensive vehicular traffic, resulting in continuous deposition of exhaust emissions and accumulation of PAHs in densely populated urban environments. Moreover, areas with frequent stop-and-go driving conditions were noted, which are known to enhance emissions of PAHs due to incomplete combustion and increased fuel consumption.

On the other hand, a second group of samples (Group B with sites 8, 12, 15 and 18) reveals a stronger influence from combustion-related emissions, potentially including diesel-powered transport sources, or other mixed combustion inputs. These locations coincide with areas affected by heavy-duty vehicle traffic and public transportation routes (Figure 1), suggesting that transport-related activities may contribute significantly to the observed HMW-PAHs (Table S2) [32,33,34,35,36,37,52]. Overall, the factor score highlighted the spatial heterogeneity of PAH contamination in Thessaloniki urban soils, with several hotspots. These patterns are consistent with the obtained diagnostic ratios and suggest that vehicular traffic emissions and fossil fuel combustion are important contributors to PAHs in the study area.

### 3.4. Carcinogenic Potential of PAHs Based on BaP-EQ Concentrations

**Figure 5:**
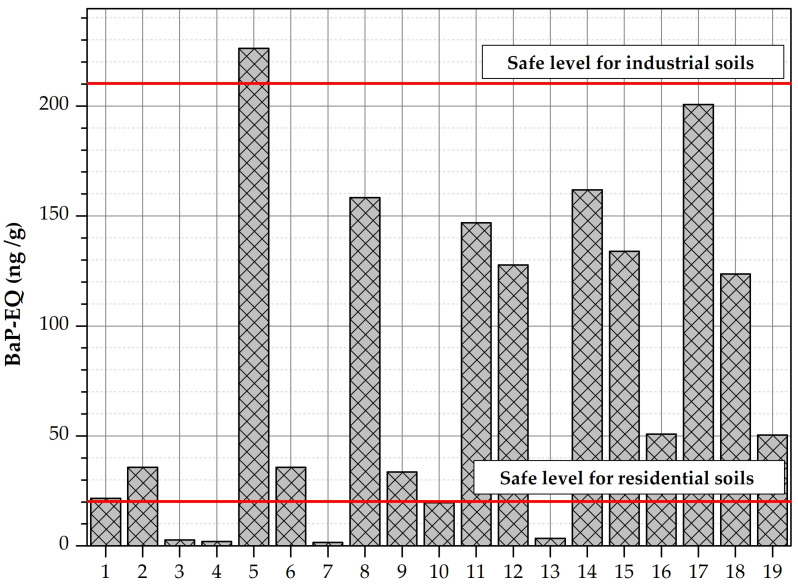
BaP-EQ of PAHs in each soil sample and the EPA’s recommended (Risk Screening Levels) safety values for residential (20 μg kg^−1^) and industrial (210 μg kg^−1^) soils.

The potential carcinogenic risk associated with PAHs in urban soils was assessed by using the benzo[a]pyrene-equivalent (BaP-EQ) approach. The varying carcinogenic potency of individual PAHs was estimated by their TEQc concentrations (in μg kg^−1^) along with the total BaP-EQ (in μg kg^−1^), and the results are given in Table S1 and Figure 5.

The calculated BaP-EQs across the studied sites show substantial variability, reflecting differences in PAH contamination intensity and source contributions. Specifically, BaP-EQ values ranged from 1.6 μg kg^−1^ to 226.2 μg kg^−1^ with an average value of 80.9 μg kg^−1^. Several sampling locations exhibit BaP-EQ concentrations exceeding the U.S. EPA’s Risk-Based Screening Level (RBSL) of 15–30 μg kg^−1^ for residential soils by almost 2.0- to 7.5-fold, indicating a potential carcinogenic risk under residential exposure scenarios, particularly for sensitive receptors such as children.

Nevertheless, a limited number of sites (samples 5 and 17) approached or exceeded the U.S. EPA’s Risk-Based Screening Level (RBSL) of 210 μg kg^−1^ for industrial soils, identifying localized hotspots of elevated carcinogenic risk. These elevated BaP-EQ values are consistent with the dominance of combustion-derived PAHs, as identified by the diagnostic ratios, and likely reflect the influence of traffic emissions and other high-temperature combustion sources in the urban environment.

Overall, the BaP-EQ assessment indicates that, while the majority of urban soils pose a low to moderate carcinogenic risk, certain areas may require further investigation and risk management, particularly in zones with residential land use or frequent human contact. It should be noted that the BaP-EQ approach provides an estimate of the relative carcinogenic potency of PAH mixtures and does not constitute a comprehensive human health risk assessment, which would require consideration of specific exposure pathways, exposure scenarios, and population-related parameters.

## 4. Conclusions

The present study provides a comprehensive assessment of the occurrence, spatial distribution, source apportionment and potential health risks of PAHs in urban soils from the city of Thessaloniki, Northern Greece. The recorded contents of PAHs (Σ_16_PAHs) ranged from 14.1 to 1565.4 μg kg^−1^ (mean 586.4 μg kg^−1^), indicating heterogeneous contamination, while 26% of the sampled soils were characterized as heavily contaminated due to levels above 1000 μg kg^−1^. Carcinogenic PAHs also exhibited significant variability (a range of 7.6–977.3 μg kg^−1^) and formed an important fraction of the total PAH burden.

PAH compositional patterns were dominated by HMW compounds (4–6 rings), indicating the prevalence of pyrogenic inputs related to high-temperature combustion processes, further supported by different isomeric diagnostic ratios. Specifically, in cross plots of Ant/(Ant + Phe) vs. Fla/(Fla + Pyr) and IP/(IP + BgP) vs. BaA/(BaA + Chr), data points were mainly clustered within the petroleum combustion field, underscoring vehicular traffic emissions as the major source of PAHs in Thessaloniki’s urban soils. Principal Component Analysis provided additional evidence for the importance of combustion-derived emissions, identifying two major source components associated with mixed urban combustion and localized diesel-related inputs. Nevertheless, these source assignments should not be considered indicative given the overlap among PAH source signatures and the limitations inherent to PCA-based source identification.

The factor score plot suggested spatial heterogeneity across the study area, reflecting the strong effect of anthropogenic and traffic-related activities. Specifically, areas closely associated with intense vehicular activity, including heavy-duty and touristic buses, traffic congestion and transport-related emissions were identified as locations characterized by relatively higher PAH concentrations.

Finally, the potential carcinogenic risk associated with PAHs in urban soils was assessed using the BaP-EQ approach, where the calculated values ranged from 1.6 μg kg^−1^ to 226.2 μg kg^−1^, revealing acceptable risk levels for most sampling sites. Limited samples (samples 5 and 17) exhibited elevated BaP-EQ values, highlighting the importance of localized anthropogenic activities in controlling human exposure potential. It should be noted that the BaP-EQ approach provides an estimate of the relative carcinogenic potency of PAH mixtures and does not constitute a comprehensive human health risk assessment.

Beyond the local assessment of urban soil contamination, this study plays a significant role in understanding the environmental behavior and sources of PAHs in urban environments, where traffic activity, tourism-related transportation, and mixed land-use patterns coexist within densely populated areas. Moreover, it contributes to the limited information available on PAHs in Mediterranean urban environments and provides one of the first comprehensive datasets for urban soils from the city of Thessaloniki. The results demonstrate that transport-related emissions remain a dominant source of PAH accumulation even in cities lacking extensive heavy industrial activity. Consequently, the findings provide valuable baseline data for future monitoring programs, support comparisons among European cities, and underline the need for targeted mitigation strategies, including pollutant accumulation control and potential human exposure reduction measures.

## Figures and Tables

**Table 1:** Summary statistics of PAH concentrations in urban soils.

Compounds		Aromatic Rings	Min	Max	Mean	Median	SD	USEPA Soil Screening Level Residential
Napthalene	Nap	2	0.15	15.85	5.00	2.90	5.03	16,000
Acenaphthylene	Ace	3	0.25	2.41	0.99	0.98	0.68	45,000
Acenaphthene	Acy	3	0.22	22.04	4.81	2.05	7.03	15,000
Fluorene	Flu	3	0.25	6.03	1.87	1.55	1.77	13,000
Phenanthrene	Phe	3	0.64	67.95	21.65	15.50	19.53	8700
Anthracene	Ant	3	0.27	28.14	10.99	9.47	9.66	3700
Fluoranthene	Fla	4	1.98	132.5	58.76	51.70	47.06	10,000
Pyrene	Pyr	4	1.69	162.9	61.61	45.70	51.92	10,000
Chrysene	Chr	4	1.71	309.8	98.99	61.70	95.84	8700
Benzo[a]anthracene	BaA	4	2.60	285.8	97.07	55.34	91.62	3700
Benzo[b,k]fluoranthene	B(b,k)F	5	1.33	180.1	69.16	50.33	57.57	1620
Benzo[a]pyrene	BaP	5	0.53	108.9	37.23	24.40	35.17	67
Indeno [1,2,3-cd]pyrene	InP	5	0.69	117.1	42.05	20.40	39.39	830
Dibenzo[a,h]anthracene	DahA	6	0.59	62.18	20.99	11.50	20.01	68
Benzo[g,h,i]perylene	BghiP	6	0.90	170.3	55.20	30.20	53.87	6800
	Σ_7_PAHs		7.59	977.3	365.5	223.3	326.2	
	Σ_16_PAHs		14.09	1565.4	586.4	365.5	515.0	

**Table 2:** Mean values of selected PAH diagnostic ratios for source apportionment.

PAH Ratio	Urban Soils Thessaloniki	Value Range	Source
∑LMW/∑HMW	0.11 ± 0.06	<1	Pyrogenic
		>1	Petrogenic
$\frac{Fla}{\left(Fla+Pyr\right)}$	0.50 ± 0.03	<0.4	Petrogenic
		0.4–0.5	Liquid fossil fuel combustion (vehicles, crude oil)
		>0.5	Biomass and coal burning
$\frac{Ant}{\left(Ant+Phe\right)}$	0.30 ± 0.12	<0.1	Petrogenic
		>0.1	Pyrogenic
$\frac{BaA}{\left(BaA+Chr\right)}$	0.53 ± 0.07	<0.2	Petrogenic
		0.2–0.35	Mixed sources
		>0.35	Pyrogenic
$\frac{InP}{\left(InP+BghiP\right)}$	0.42 ± 0.10	<0.2	Petrogenic
		0.2–0.5	Liquid fossil fuel, vehicle emission
		>0.5	Grass, wood and coal combustion
BaP/BghiP	0.65 ± 0.18	≤0.6	Traffic emissions
		>0.6	Non-traffic emissions

**Table 3:** Main group components derived by Principal Component Analysis (PCA). Values in bold represent a strong correlation in specific components, while values underlined indicate a moderate correlation.

	PC1	PC2
Nap	**0.898**	0.410
Ace	**0.924**	0.165
Acy	−0.022	**0.996**
Flu	**0.992**	0.022
Phe	**0.964**	0.243
Ant	**0.783**	0.581
Fla	**0.731**	0.667
Pyr	**0.855**	0.508
Chr	**0.927**	0.370
BaA	**0.895**	0.440
B(b,k)F	0.530	**0.840**
BaP	**0.898**	0.435
InP	0.486	**0.872**
DhA	**0.897**	0.437
BP	**0.907**	0.402
Eigenvalue	12.935	1.754
Explained variance (%)	86.24	11.70
Cumulative (%) of variance	86.24	97.94

## Data Availability

Data that support the findings of this study are available from the corresponding author upon reasonable request.

## Supplementary Materials

The following supporting information can be downloaded at: https://www.mdpi.com/article/10.3390/toxics14070582/s1. Table S1. Toxic equivalent (TEQ) and total BaP equivalent (BaP-EQ) concentrations (μg kg^−1^) of PAHs in urban soils from Thessaloniki (Northern Greece). Table S2. PAHs concentrations (μg kg^−1^) in urban soils from Thessaloniki, Northern Greece along with bibliographic data regarding PAHs levels in multiple urban agglomerations.
