# Supplementary material for: Occurrence, Source Apportionment and Health Risk Potential of Polycyclic Aromatic Hydrocarbons (PAHs) in Urban Soils from Thessaloniki City (Northern Greece): A Case Study
Source: Toxics. 2026 Jul 1;14(7):582. doi: 10.3390/toxics14070582 (PMC13417453; doi:10.3390/toxics14070582)
Supplement: Supplementary file 1 [file toxics-14-00582-s001.zip › toxics-4371052-supplementary.pdf]

Occurrence, source apportionment and health risk potential of polycyclic aromatic hydrocarbons (PAHs) in urban soils from Thessaloniki city (Northern Greece): a case study

Anna Bourliva[1] *, Evangelia E. Golia[1], Evangelos Bakeas[2], Konstantinos Koukoulakis[2], and Ioannis Papadopoulos[1]

[1] Soil Science Laboratory, School of Agriculture, Faculty of Agriculture, Forestry and Natural Environment, Aristotle University of Thessaloniki, University campus, 541 24 Thessaloniki, Greece

[2]Environmental Chemistry Laboratory, Department of Chemistry, National and Kapodistrian University of Athens, Zografou 157 84, Greece

*Corresponding author: A. Bourliva, tel: +302310 998809, email: annab@geo.auth.gr

Table S1. Toxic equivalent (TEQ) and total BaP equivalent (BaP-EQ) concentrations (μg kg-1) of PAHs in urban soils from the city of Thessaloniki (Northern Greece).

Sample	*NA*	*ACE*	*ACY*	*FL*	*PH*	*ANC*	*FLA*	*PY*	*CH*	*BaA*	*B(b,k)F*	*BaP*	*IP*	*DhA*	*BP*	*BaP-EQ*
TEF	*0.001*	*0.001*	*0.001*	*0.001*	*0.001*	*0.01*	*0.001*	*0.001*	*0.01*	*0.1*	*0.1*	*1*	*0.1*	*1*	*0.01*	
**1**	0.001	0.000	0.001	0.001	0.008	0.012	0.021	0.020	0.165	2.279	3.309	9.139	1.248	5.262	0.154	**21.6**
**2**	0.000	0.001	0.000	0.002	0.018	0.039	0.053	0.052	0.609	4.238	5.356	13.161	1.830	10.200	0.127	**35.7**
**3**	0.000	0.000	0.000	0.000	0.002	0.004	0.003	0.003	0.027	0.441	0.451	0.872	0.187	0.743	0.040	**2.8**
**4**	0.000	0.000	0.000	0.000	0.002	0.003	0.002	0.002	0.017	0.308	0.194	0.732	0.087	0.615	0.022	**2.0**
**5**	0.016	0.002	0.002	0.006	0.068	0.281	0.133	0.163	3.098	28.576	13.084	108.915	7.978	62.183	1.703	**226.2**
**6**	0.002	0.001	0.002	0.001	0.013	0.095	0.026	0.026	0.440	3.975	2.834	17.105	2.009	8.926	0.227	**35.7**
**7**	0.000	0.000	0.000	0.000	0.001	0.003	0.002	0.002	0.018	0.260	0.133	0.534	0.069	0.586	0.009	**1.6**
**8**	0.009	0.001	0.022	0.002	0.031	0.221	0.128	0.116	1.757	18.179	18.007	68.762	11.713	38.482	0.967	**158.4**
**9**	0.002	0.001	0.001	0.001	0.009	0.030	0.037	0.033	0.456	4.229	3.562	16.301	1.804	6.904	0.215	**33.6**
**10**	0.000	0.000	0.001	0.001	0.008	0.012	0.016	0.019	0.155	2.200	2.960	8.700	1.020	4.400	0.119	**19.6**
**11**	0.010	0.002	0.002	0.004	0.044	0.178	0.089	0.106	2.015	19.160	8.750	69.650	5.360	40.400	1.169	**146.9**
**12**	0.007	0.001	0.017	0.002	0.026	0.182	0.100	0.092	1.339	15.140	14.370	54.500	9.430	31.700	0.767	**127.7**
**13**	0.001	0.000	0.000	0.000	0.003	0.011	0.003	0.003	0.020	0.470	0.320	1.330	0.112	1.100	0.029	**3.4**
**14**	0.012	0.002	0.002	0.004	0.043	0.203	0.090	0.112	2.050	19.950	9.950	77.930	5.460	44.800	1.219	**161.8**
**15**	0.008	0.001	0.016	0.002	0.026	0.187	0.104	0.094	1.356	14.370	13.960	58.900	10.180	33.800	0.898	**133.9**
**16**	0.003	0.001	0.003	0.002	0.016	0.143	0.035	0.039	0.617	5.534	3.940	24.400	2.970	12.770	0.302	**50.8**
**17**	0.013	0.002	0.003	0.006	0.059	0.233	0.119	0.144	2.667	25.030	10.950	98.500	7.040	54.500	1.398	**200.7**
**18**	0.007	0.001	0.017	0.001	0.023	0.197	0.104	0.100	1.356	14.050	14.240	53.400	9.350	29.990	0.784	**123.6**
**19**	0.003	0.002	0.003	0.002	0.014	0.054	0.052	0.046	0.645	6.050	5.033	24.600	2.040	11.500	0.339	**50.4**

Table S2. PAHs concentrations (μg kg^{-1}) in the urban soils from the city of Thessaloniki, Northern Greece along with bibliographic data regarding PAHs levels in multiple urban agglomerations.

Sample	***NA***	***ACY***	***ACE***	***FL***	***PH***	***ANC***	***FLA***	***PY***	***CH***	***BaA***	***B(b,k)F***	***BaP***	***IP***	***DhA***	***BP***	***Σ_7PAHs***	***Σ_{16}PAHs***	
**1**	1.25	0.65	0.35	0.60	8.06	1.19	20.73	19.93	16.49	22.79	33.09	9.14	12.48	5.26	15.43	**99.25**	**167.42**	
**2**	0.41	0.44	0.70	1.73	18.35	3.92	53.37	52.44	60.92	42.38	53.56	13.16	18.30	10.20	12.66	**198.52**	**342.54**	
**3**	0.27	0.26	0.25	0.27	1.90	0.40	3.15	2.70	2.69	4.41	4.51	0.87	1.87	0.74	3.99	**15.11**	**28.32**	
**4**	0.37	0.22	0.26	0.34	2.28	0.31	2.15	1.98	1.71	3.08	1.94	0.73	0.87	0.61	2.23	**8.95**	**19.10**	
**5**	15.85	2.47	2.41	5.60	67.95	28.14	132.53	162.91	309.80	285.76	130.84	108.92	79.78	62.18	170.28	**977.28**	**1565.40**	
**6**	2.26	2.05	0.53	1.21	13.07	9.47	25.87	25.73	44.01	39.75	28.34	17.11	20.09	8.93	22.71	**158.23**	**261.13**	
**7**	0.28	0.23	0.26	0.25	0.64	0.27	1.98	1.69	1.85	2.60	1.33	0.53	0.69	0.59	0.90	**7.59**	**14.09**	
**8**	9.38	22.04	0.98	1.83	31.23	22.08	127.93	116.41	175.69	181.79	180.07	68.76	117.13	38.48	96.71	**761.92**	**1190.52**	
**9**	1.64	1.41	0.62	0.68	8.62	3.01	36.83	33.14	45.65	42.29	35.62	16.30	18.04	6.90	21.47	**164.80**	**272.22**	
**10**	0.15	0.66	0.34	0.61	7.70	1.20	15.70	18.80	15.50	22.00	29.60	8.70	10.20	4.40	11.90	**90.40**	**147.46**	
**11**	9.90	1.67	1.61	4.01	43.66	17.80	89.30	105.60	201.50	191.60	87.50	69.65	53.60	40.40	116.90	**644.25**	**1034.70**	
**12**	7.44	16.70	1.10	1.55	25.60	18.20	100.40	92.30	133.90	151.40	143.70	54.50	94.30	31.70	76.70	**609.50**	**949.49**	
**13**	0.61	0.33	0.36	0.42	3.20	1.10	2.60	2.70	1.99	4.70	3.20	1.33	1.12	1.10	2.90	**13.44**	**27.66**	
**14**	12.00	2.23	1.80	4.22	43.40	20.30	90.44	111.70	205.00	199.50	99.50	77.93	54.60	44.80	121.90	**681.33**	**1089.32**	
**15**	8.01	15.55	1.10	1.56	25.66	18.70	103.50	94.20	135.60	143.70	139.60	58.90	101.80	33.80	89.80	**613.40**	**971.48**	
**16**	3.10	2.66	1.10	1.67	15.50	14.30	34.50	39.20	61.70	55.34	39.40	24.40	29.70	12.77	30.20	**223.31**	**365.54**	
**17**	12.50	2.50	2.14	6.03	58.50	23.30	119.40	143.80	266.70	250.30	109.50	98.50	70.40	54.50	139.80	**849.90**	**1357.87**	
**18**	6.70	16.80	1.10	1.30	22.56	19.70	104.30	99.70	135.60	140.50	142.40	53.40	93.50	29.99	78.40	**595.39**	**945.95**	
**19**	2.90	2.55	1.77	1.60	13.50	5.40	51.70	45.70	64.50	60.50	50.33	24.60	20.40	11.50	33.90	**231.83**	**390.85**	
																		**References**
**Santiago de Compostela, Spain**	7.4	2.1	1.6	1.8	27.1	5.2	72.8	58.2	59.7	39.1		43.1	30.7		32		**458**	[33]
**Novi Sad, Serbia**	9	1.8	1.5	2.8	36	4.4	61	49	44	20	52	22	20	6	33		**363**	[35]
**Krakow, Poland**	31.8	107	53.4	148	2,145	531	3,909	2,980	2,120	2,481	1,670	1,302			307	**7947.00**	**18,220**	[36]
**Terragona, Spain**	21.2	3	1.9	nd	37.8	7.5	69.2	58	34	27.3	69	35.2	35.2	6.3	31.3	**206.9**	**437.7**	[32]
**London, UK**	340	410	440	660	2,900	1,000	12,000	11,000	5,600	8,700		6,900	6,800	910	6,200		**68,000**	[52]
**Glasgow, UK**	132		92.7	110	1951	254	1729	1,763	1,150	920	1,207	971	684	97	867		**11,927.7**	
**Ljubljana, Slovenia**	28.4		9.5	12.4	209	13.7	135	104	79.1	61.7	107.5	76.8	74.6	11.9	65		**988.6**	[37]
**Torino, Italy**	31.8		14.3	23.7	227	32.3	272	289	194	162	252	229	85.6	19.6	156		**1,988.3**	
**Beijing, China (urban parks)**	18	5	3	8	43	17	63	61	53	35	80	29	20	7	28	**219**	**460**	[34]
**Residential SSL**	16,000	45,000	15,000	13,000	8,700	3,700	10,000	10,000	8,700	3,700	1,620	67	830	68	6,800			
